# Comparative Analysis of *SMN2* Splicing Activity and Protein Production Following In Vitro Treatment with the Generic Risdiplam Drug Vapromin^®^ and the Reference Drug Evrysdi^®^

**DOI:** 10.3390/biomedicines14091994

**Published:** 2026-09-04

**Authors:** Olga Strizhakova, Andrei Pershin, Yana Bahareva, Aleksandr Kazarov, Ivan Lyagoskin, Evgenia Bocharova, Roman Anisimov, Yury Gladchenko, Natalia Kholod, Inessa Kirik, Anna Gudilina, Rakhim Shukurov, Ravil Khamitov

**Affiliations:** JSC “GENERIUM”, 14, Vladimirskaya Street, Volginskiy 601125, Vladimir Region, Russia; strizhakova@ibcgenerium.ru (O.S.); kazarov@ibcgenerium.ru (A.K.);

**Keywords:** SMA, SMN, risdiplam, fibroblast, generic drug, Exon 7 inclusion, biologic activity, reporter cell line

## Abstract

**Background/Objectives**: Risdiplam is a low-molecular-weight small-molecule modifier of *SMN2* pre-mRNA splicing that was developed for spinal muscular atrophy (SMA) therapy and approved for the treatment of SMA as Evrysdi^®^ (Roche, Basel, Switzerland). Vapromin^®^ is a generic drug produced by JSC GENERIUM. In order to evaluate its biological activity and compare different batches of the reference drug and generic risdiplam, we performed comprehensive *in vitro* procedures. It is important to emphasize that this study did not assess the bioequivalence of the medicinal products for the purpose of comparing the biopharmaceutical quality of the generic and reference products. Instead, this study was designed specifically to compare their biological activity using cells derived from SMA patients and a reporter cell line. **Methods**: The biological activity of generic risdiplam was compared with that of the reference drug by assessing increases in SMN protein production and the relative transcription level of *SMN2* mRNA in fibroblasts from SMA donors. Additionally, Exon 7 inclusion efficiency was evaluated using a constructed reporter cell line. **Results**: Using primary dermal fibroblasts from SMA probands, we demonstrated a concentration-dependent relationship between risdiplam concentration and *SMN2* FL and *SMN2* Δ7 transcript levels. At a risdiplam concentration of 0.18 μM, the relative *SMN2* full-length transcript levels reached a maximum, with a mean 2.5-fold increase observed for both Evrysdi^®^ (Roche, Basel, Switzerland) and Vapromin^®^. In primary fibroblasts derived from three SMA patients, the 2 SD quality range for Evrysdi^®^ (Roche, Basel, Switzerland) was 98.0–106.7% (σ(log RP) = 0.0092), and the activities of all Vapromin^®^ batches fell within this range. Using the reporter cell line, the quality range for Evrysdi^®^ (Roche, Basel, Switzerland) was 88.32–117.3% (σ = 0.0309), and the Vapromin^®^ values also fell within this range. **Conclusions**: This study experimentally confirmed that the in vitro biological activity of the generic drug Vapromin^®^ is comparable to that of Evrysdi^®^ (Roche, Basel, Switzerland). Further research should be conducted to confirm bioequivalence between the two products.

## 1. Introduction

Spinal muscular atrophy (SMA) is a recessively inherited neuromuscular disorder caused by a deficiency in the survival motor neuron (SMN) protein due to a mutation in the *SMN1* gene on chromosome 5q, leading to generalized symmetrical muscle weakness. SMA has an estimated prevalence of 1 to 2 per 100,000 individuals and an incidence of approximately 1 in 10,000 live births [1]. According to Jeong et al. [2], the global lifetime Bayesian prevalence of SMA across all ages was 0.010% (95% credible interval, 0.002–0.021). The clinical manifestation of the disease is highly variable. Patients with SMA are typically divided into four subgroups based on the age of disease onset and the developmental motor milestones achieved. In its most severe form, SMA is the leading genetic cause of infant mortality [3,4].

In addition to the *SMN1* gene, the human genome also contains 1–8 copies of the centromeric survival motor neuron gene (*SMN2*), located on the same chromosome 5. Due to alternative pre-mRNA splicing, which predominantly generates mRNA transcripts lacking exon 7, expression of the *SMN2* gene results in approximately 80–90% truncated, unstable/non-functional SMNΔ7 protein and only 10–20% full-length SMN protein, which is able to partially compensate for the absence of functional *SMN1* expression in SMA. Therefore, a higher *SMN2* copy number correlates with milder SMA phenotypes [5].

Currently, SMA remains incurable; however, progress has been made in the development of therapeutic approaches, including:

(1) Transplantation of intrathecal stem cells [6];

(2) Administration of exogenous *SMN1* via recombinant viral vectors [7];

(3) Upregulation of *SMN2* expression using low-molecular-weight histone deacetylase inhibitors [8,9];

(4) Correction of *SMN2* splicing using antisense oligonucleotides and low-molecular-weight compounds to promote exon 7 inclusion in *SMN2* mRNA transcripts, thereby increasing the production of full-length SMN protein [10,11,12]. Nevertheless, the long-term real-world effectiveness, safety, and differential impact of these approaches and registered drugs across SMA phenotypes remain areas of active investigation [1].

Risdiplam is a low-molecular-weight modifier of *SMN2* pre-mRNA splicing developed for SMA therapy and approved for the treatment of SMA as Evrysdi^®^ (Roche, Basel, Switzerland). Risdiplam received approval from the European Medicines Agency (EMA) in March 2021 based on pivotal clinical trials that established its efficacy and safety in both infantile-onset (Type 1) and later-onset (Types 2 and 3) SMA across a broad pediatric and young adult population. Further data on the efficacy, safety, pharmacokinetics, and pharmacodynamics of risdiplam can be found in the comprehensive review by Belančić et al. [1].

Switching SMA patients from another drug, nusinersen, to risdiplam has demonstrated effectiveness (non-inferiority), safety, and tolerability in a heterogeneous pediatric-and-adult “switch” cohort. This is expected to further improve the quality and standards of care, as well as the safety profile, for a notable proportion of SMA patients, particularly those who require a switch from nusinersen to other disease-modifying therapies (DMTs) for clinical or personal reasons [13].

Risdiplam has been reproduced by JSC GENERIUM under the brand name Vapromin^®^ (GNR-115). Generic drugs are considered clinically equivalent to brand-name drugs when they demonstrate comparable safety and effectiveness. The use of generic pharmaceutical products is promoted to reduce costs and increase access to healthcare [14]. However, rigorous quality and safety assessments of pharmaceutical products are essential to achieve these goals. According to the EMA, a proposed generic medicinal product must demonstrate equivalence in biopharmaceutical quality to the corresponding reference medicinal product to enable bridging of the preclinical and clinical data generated for the reference product. The EMA therefore provides detailed requirements for the design, conduct, and evaluation of bioequivalence studies.

In the present study, we focused exclusively on evaluating the *in vitro* biological activity of the proposed generic risdiplam product and did not assess its bioequivalence or physicochemical characteristics. To evaluate biological activity, we performed a comparative analysis of the reference and generic drugs at two levels: SMN protein production and *SMN2* mRNA transcript levels in treated fibroblasts derived from SMA donors. Additionally, exon 7 inclusion efficiency was assessed using a constructed reporter cell line.

## 2. Materials and Methods

### 2.1. Donors

Skin samples were obtained from three donors of European descent diagnosed with SMA (probands 1, 2, and 3, aged 38, 29, and 40 years, respectively). Skin biopsies were performed at a local medical facility under local anesthesia. The samples were delivered to the laboratory within 24 h of collection. The main inclusion criteria were a confirmed diagnosis and patient safety. According to multiplex ligation-dependent probe amplification (MLPA), all three donors harbored a homozygous deletion of exons 7 and 8 in the *SMN1* gene. Probands 1 and 2 had three copies of the *SMN2* gene, whereas proband 3 had four copies.

### 2.2. Processing Skin Fibroblasts

Skin samples, 3 mm in diameter, were delivered to the laboratory in cooled DMEM/F12 medium (PanEco, Moscow, Russia) containing a 1:100 solution of penicillin/streptomycin/amphotericin B (HiMedia, Mumbai, Maharashtra, India).

Mechanically minced biopsies were enzymatically dissociated in DMEM/F12 supplemented with 5% fetal bovine serum (Capricorn Scientific GmbH, Ebsdorfergrund, Germany), 0.2% dispase (Corning Inc., Corning, NY, USA), and 0.1 mg/mL collagenase type I (Thermo Fisher, Waltham, MA, USA) for 2 h. The cell suspension was filtered through a 70 μm cell strainer (Wuxi NEST Biotechnology Co., Ltd., Wuxi City, China).

Dissociated cells were pelleted at 200g and resuspended in DMEM/F12 medium containing 15% non-inactivated fetal bovine serum (HyClone, Logan, UT, USA), penicillin/streptomycin/amphotericin B, and 2 mM L-glutamine (Lonza Group, Basel, Switzerland). Cells were passaged upon reaching 90% confluence of the monolayer. Cell viability was determined by staining with a 0.4% trypan blue solution (PanEco, Moscow, Russia).

### 2.3. Calculation of the Fibroblast Population Doubling Time

Fibroblasts were seeded at a density of 1.5 × 10^4^ cells per 1 cm^2^ at each passage and cultured until they reached 90% confluence. Cells were detached from the plastic surface by trypsinization. Cell counting was performed using a Countess 3 automated cell counter (Thermo Fisher, Waltham, MA, USA). The population doubling time was calculated using the following formula:

$$T_{2}=\frac{T_{k}*ln(2)}{ln(\frac{N_{k}}{N_{0}})},$$

where *T*_2_—doubling time;

*T*_k_—the duration of one passage in hours;

*N*_k_—the final number of cells harvested after *T*_k_;

*N*_0_—the initial number of cells seeded;

ln—natural logarithm, ln (2) −0.693.

### 2.4. Cytotoxicity Assay

One hundred microliters of a cell suspension (6 × 10^5^ cells/mL) was seeded into each well of a 96-well plate and incubated for 24 h in a cell culture incubator (37 °C, 5% CO_2_). Subsequently, different concentrations of risdiplam (0.3 nM to 2.2 μM), prepared in culture medium, were added to the wells in triplicate, and the cells were incubated for an additional 20 h under the same conditions. Untreated wells containing culture medium alone served as controls. After a total exposure time of 44 h, XTT reagent (HiMedia, Mumbai, Maharashtra, India; Cat. No. CCK015) was added according to the manufacturer’s instructions. Optical density was measured at 492/690 nm using a spectrophotometer. Cytotoxicity was expressed as the percentage ratio of the absorbance of treated wells to that of the untreated control.

### 2.5. Drug Treatment of SMA Fibroblasts for In Vitro Evaluation of Exon 7 Inclusion Efficiency in SMN2 mRNA Splicing

Fibroblast suspensions from three SMA donors were added to the wells of a 96-well culture plate (Costar, Cambridge, MA, USA; Cat. No. 3599) at 100.0 μL/well and a concentration of 0.6 × 10^6^ cells/mL in culture medium. The cells were incubated at 37 ± 1 °C and a CO_2_ concentration of 5.0 ± 0.5%. Serial dilutions of risdiplam batches, at concentrations ranging from 0.0003 to 2.2 µM in cell culture medium, were added to the wells containing primary fibroblasts.

Cells were treated for 48 h under the same conditions. The Evrysdi^®^ (Roche, Basel, Switzerland) batch B1056B11 was used as the reference standard (RS) for the calculation of relative activity. Independent serial dilutions of the RS were prepared for each plate. Wells containing cells and cell culture medium alone served as negative controls.

After incubation, the cells were washed three times with serum-free DMEM/F12 and twice with phosphate-buffered saline to remove residual medium. Subsequently, 100 μL of distilled water was added to each well to lyse the cells. Completion of lysis was monitored under a microscope using a 40× objective lens. The lysates were clarified by centrifugation at 1000 rpm for 5 min.

### 2.6. Total Protein and SMN Concentration Measurement

The concentration of total soluble protein in the samples was determined using a BCA Protein Assay Kit (#23227, Pierce BCA Protein Assay Kit; Thermo Fisher, Waltham, MA, USA). Samples were normalized to 1 g of total soluble protein based on the BCA analysis.

SMN protein levels in fibroblasts were quantified using an ELISA kit (#ADI-900-209, Enzo Life Sciences, Farmingdale, NY, USA) and expressed as nanograms per gram of total protein, according to previously described procedures [15,16].

### 2.7. RT-qPCR Analysis of Full-Length SMN2 mRNA and Exon 7 Deleted SMN2 mRNA

Total RNA from treated fibroblasts was isolated using the AllPrep DNA/RNA Kit (QIAGEN, Venlo, The Netherlands) and stored at −80 °C.

The mRNAs of *SMN2* FL, *SMN2* Δ7, and *GAPDH* were quantified using a single-tube RT-qPCR mix (Gen Terra, Moscow, Russia) and specific primers described in [17]:

*SMN2* FL forw—GCTCACATTCCTTAAATTAAGGAGAAA;

*SMN2* FL rev/*SMN2* Δ7 rev—TCCAGATCTGTCTGATCGTTTCTT;

*SMN2* Δ7 forw—TGGCTATCATACTGGCTATTATATGGAA;

*GAPDH* forw—CAACGGATTTGGTCGTATTGG;

*GAPDH* rev—TGATGGCAACAATATCCACTTACC.

RT-qPCR was carried out at the following temperatures for the indicated times: Step 1 (cDNA synthesis): 55 °C (15 min); Step 2: 95 °C (10 min); Step 3: 95 °C (15 s); and Step 4: 60 °C (1 min); Steps 3 and 4 were repeated for 40 cycles.

The Ct values for each mRNA were converted to mRNA abundance using the actual PCR efficiencies. *SMN2* FL and Δ7 mRNAs were normalized to *GAPDH* and to the untreated controls and plotted as fold change relative to the control treatment.

### 2.8. Construction of a Reporter Cell Line Producing Luciferase upon Exon 7 Inclusion

The CHO SMN2-LUC MP 4G6 reporter cell line (JSC GENERIUM) was generated to evaluate the effectiveness of *SMN2* mRNA splicing modifiers as follows: CHO cells were stably transformed with an expression cassette encoding a chimeric protein consisting of *SMN2* gene exons 1–8 and introns 6 and 7, fused in a single reading frame with the firefly luciferase gene under the control of the native *SMN2* promoter. The cell line produces luciferase upon successful inclusion of exon 7 in mature *SMN2* mRNA. Thus, the luminescence intensity following the addition of the D-luciferin substrate is directly proportional to the ability of the drug to correct alternative splicing.

### 2.9. Determination of Exon 7 Inclusion Efficiency Using CHO SMN2-LUC MP 4G6 Reporter Cell Line

Different concentrations (0–60.2 µM) of the evaluated batches of Evrysdi^®^ (Roche, Basel, Switzerland) and Vapromin^®^ in cell culture medium (BalanCD, 94120, FUJIFILM Irvine Scientific, Santa Ana, CA, USA) were added to the wells in triplicate and incubated for 20 h in a cell culture incubator (37 °C, 5% CO_2_). Subsequently, 50 µL of luciferase substrate (G7940, Promega, Madison, WI, USA) was added to detect luminescence.

The specific activity of each test sample relative to the reference standard (RS) was determined using a four-parameter logistic function with GraphPad Prism 8.0 software (GraphPad Software, Inc.).

### 2.10. Western Blot

Fibroblasts from a healthy donor, an SMA patient, and an SMA patient treated with risdiplam were washed three times with serum-free medium and ice-cold phosphate-buffered saline (PBS). Cells were lysed on ice for 30 min in CHAPS-based lysis buffer (CHAPS BioChemica, Applichem GmbH, Darmstadt, Germany, A1099.0050) supplemented with phenylmethanesulfonyl fluoride (PMSF; Servicebio, Wuhan, China GC207002-5G). The total protein concentration in the cleared cell lysates was determined using the Bradford assay (Coomassie [Bradford] Protein Quantitative Assay Kit, Servicebio, Wuhan, China, G2001-250ML). Lysates were mixed with 4× SDS sample buffer containing β-mercaptoethanol and heated at 95 °C for 10 min. Equal amounts of protein (23 µg per lane) were separated by electrophoresis on a 10% SDS-polyacrylamide gel at 200 V for 50 min. Proteins were subsequently transferred onto methanol-activated polyvinylidene fluoride (PVDF) membranes using a semi-dry transfer system. Immediately after transfer, the membranes were blocked with 3% non-fat dry milk in Tris-buffered saline containing Tween 20 (TBST) for 1 h at room temperature. The PVDF membranes were then cut into strips and incubated overnight at 4 °C with mouse monoclonal antibodies against β-actin (1:1000; Servicebio, Wuhan, China, GB15689-100) or SMN (1:500; Invitrogen, Carlsbad, CA, USA, MA1-5878). Following washing with PBST, the membranes were incubated with horseradish peroxidase (HRP)-conjugated anti-mouse secondary antibody (1:1000; Dako, Agilent Technologies, Santa Clara, CA, USA, P0260) for 2 h at room temperature. The membranes were then washed extensively with PBST, and the immunoreactive bands were visualized using an enhanced chemiluminescence (ECL) substrate for HRP (Advansta, San Jose, CA, USA K-12045-D20). Molecular weight marker—Biolabmix RAV-10 (Novosibirsk, Russia, PS-1050) 6.5 to 270 kDa (6.5, 16, 30, 37, 52, 66, 95, 130, 175, 270 kDa).

### 2.11. Statistical Evaluation

A quality range (QR) approach was employed to evaluate the comparability of generic risdiplam (Vapromin^®^) with the reference product (Evrysdi^®^ (Roche, Basel, Switzerland)) [18,19].

The QR limits were defined based on the variation observed in the reference product, calculated as the mean ± 2 standard deviations (SDs) according to the formula:

µ(Log _RP_) ± 2 × σ(Log _RP_),

where:

µ(Log _RP_)—arithmetic mean of the log-transformed values of % RSA obtained from the Evrysdi (Roche, Basel, Switzerland) LP^®^ batches;

σ(Log _RP_)—standard deviation of the log-transformed values obtained from the Evrysdi (Roche, Basel, Switzerland) LP^®^ batches.

To assess the comparability of the drugs in terms of specific activity, the quality range (QR) approach was used, as described in the FDA guidance “Development of Therapeutic Protein Biosimilars: Comparative Analytical Assessment and Other Quality-Related Considerations” (see [18,19] for details). Comparability was considered confirmed if the %RSA values obtained for individual batches of Vapromin^®^ fell within the QR established for Evrysdi^®^ (Roche, Basel, Switzerland). The QR was determined as follows: the %RSA values for individual batches of Evrysdi^®^ (Roche, Basel, Switzerland) were log-transformed; the arithmetic mean (M) and standard deviation (SD) were calculated; and the M ± 2SD limits were back-transformed to the original %RSA scale.

SMN protein level versus drug log-concentration curves were fitted using a linear trend. The extra sum-of-squares F-test was used to assess the significance of the trend (i.e., whether the slope differed from zero) and to compare trend parameters between the drugs.

We were unable to identify a suitable function that accurately fitted the relationship between mRNA transcription levels and either drug concentration or log-concentration. Therefore, the correlation was assessed using Spearman’s r. The area under the transcription level curves (AUC), as well as the standard error of the AUC (SE of AUC), was calculated using the trapezoidal rule and DeLong’s method, respectively, and then compared between the drugs using the Z-test.

An unpaired Student’s *t*-test was used to compare the population doubling times of dermal fibroblasts from presumably healthy donors and SMA probands.

All calculations were performed using GraphPad Prism 8.0 and Microsoft Excel. A significance level of 0.05 was used throughout the analysis.

## 3. Results

Fibroblasts are the most relevant primary cell model for SMA studies, aside from neuronal cells, and are widely used in pathophysiological investigations. This study shows using fibroblasts from 3 SMA probands and healthy donors.

Dermal fibroblast cultures were established via enzymatic dissociation of skin biopsy samples. The cells adhered tightly to the plates and formed growth foci after 3–4 days of maintenance. Adherent cells acquired an elongated shape, with flattened cytoplasm. The doubling time of skin fibroblast cultures obtained from healthy donors did not differ significantly from that of SMA probands (*p* value = 0.5638). However, cells from SMA patients exhibited slower growth over several passages compared to cells from healthy donors. *SMN2* expression in SMA proband skin fibroblasts was significantly lower than in healthy donors (*p* value 0.0015).

Three batches of Vapromin^®^ and three batches of the original drug Evrysdi^®^ (Roche, Basel, Switzerland) were analyzed in order to compare their biological properties.

### 3.1. Relative Transcription Level of SMN2 FL and SMN2 Δ7 in SMA Donors’ Fibroblasts

**Figure 1:**
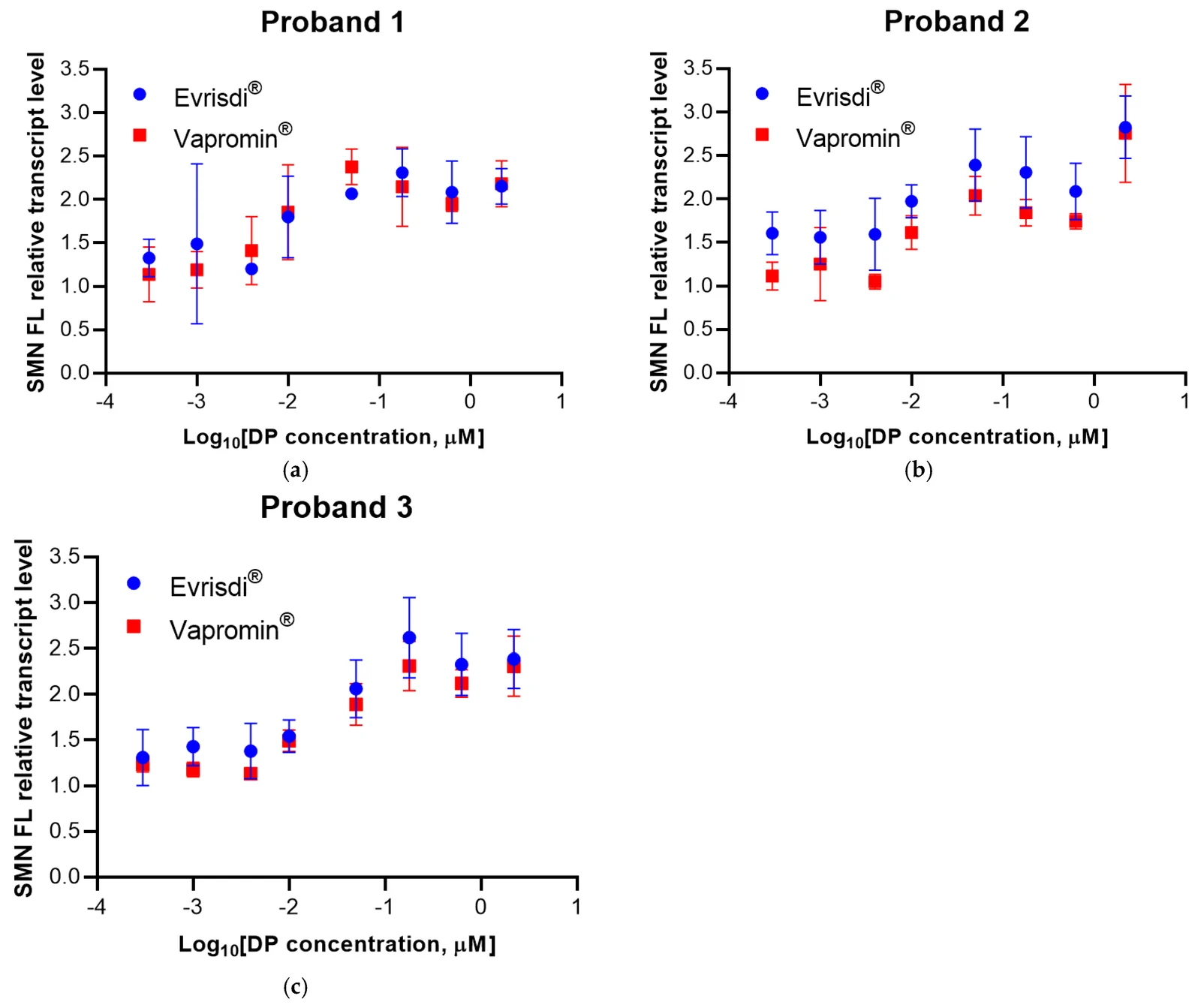
Plots of *SMN2* FL mRNA transcription levels versus each drug log-concentration in fibroblasts from three SMA probands (**a**–**c**). Transcription levels are expressed as fold changes relative to the baseline level in fibroblasts from SMA probands without drug. Mean transcription levels and standard deviations (based on triplicates) are shown for each drug concentration.

**Figure 2:**
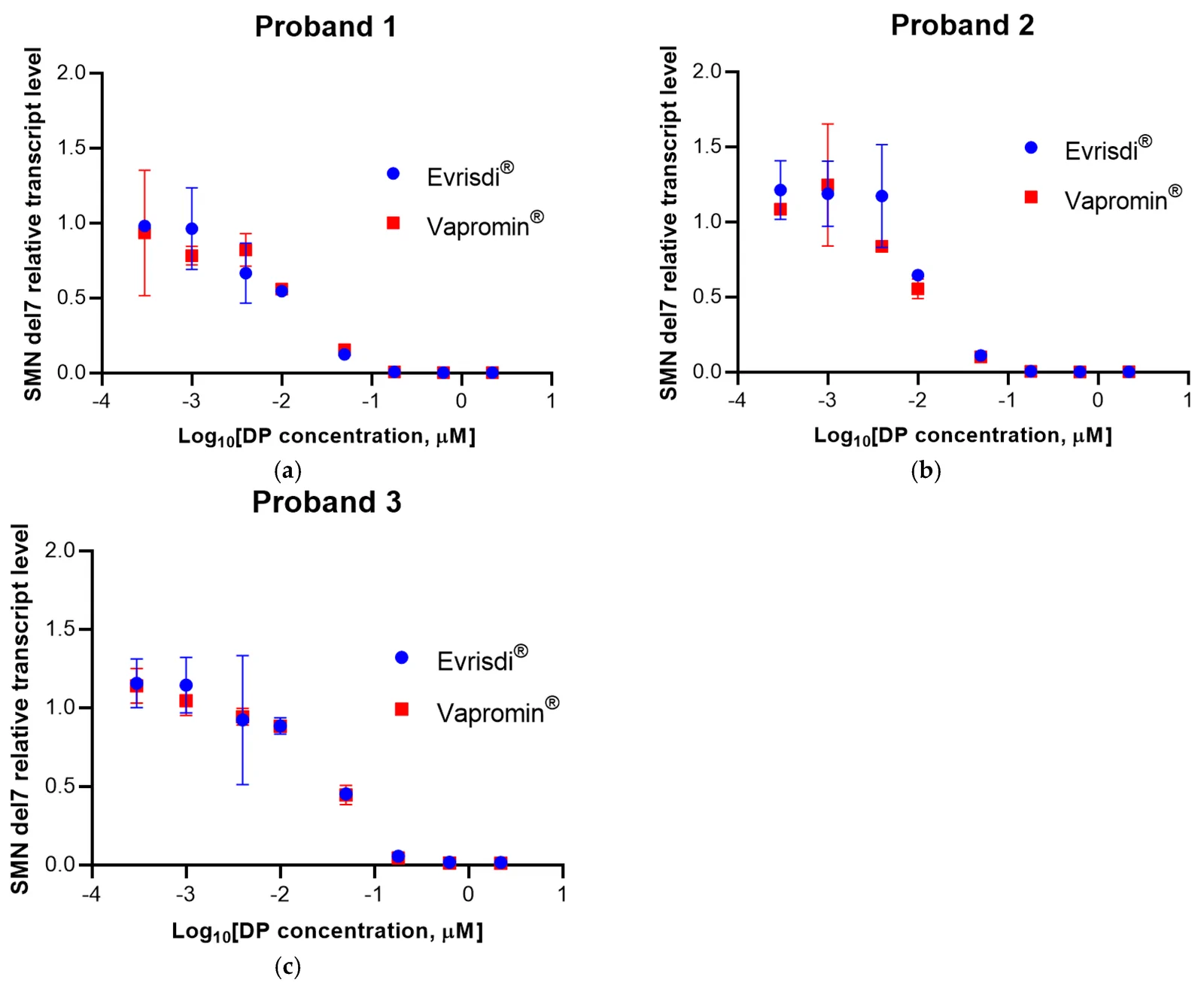
Plots of *SMN2* Δ7 mRNA transcription levels versus each drug log-concentration in fibroblasts from three SMA probands (**a**–**c**). Transcription levels are expressed as fold changes relative to the baseline level in fibroblasts from SMA probands without drug. Mean transcription levels and standard deviations (based on triplicates) are shown for each drug concentration.

Fibroblasts from three SMA probands were exposed to increasing concentrations (0.0003 to 2.2 µM) of risdiplam preparations (either Evrysdi^®^ (Roche, Basel, Switzerland) or Vapromin^®^), followed by measurement of the relative transcription levels of *SMN2* FL and *SMN2* Δ7, expressed as fold changes relative to the baseline level in fibroblasts from SMA probands without drugs (Figure 1 and Figure 2).

Spearman’s r showed a significant correlation (positive for *SMN2* FL; negative for *SMN2* Δ7) between the transcription level and each drug log-concentration. Area under each curve (AUC) values were calculated, and the Z-test showed no significant differences in AUC between the drugs for each proband (Table 1 and Table 2).

A relationship between risdiplam concentration and the transcription levels of *SMN2* FL and *SMN2* Δ7 was established. Addition of 0.18 μM risdiplam increased the relative transcription level of *SMN2* FL by 2.5 times on average for both Evrysdi^®^ (Roche, Basel, Switzerland) and Vapromin^®^. Simultaneously, the relative transcription level of *SMN2* Δ7 decreased to 0 for all samples.

These data allow us to conclude that Evrysdi^®^ (Roche, Basel, Switzerland) and Vapromin^®^ have similar effects on the relative transcription levels of *SMN2* FL and *SMN2* Δ7 in the fibroblasts of SMA donors.

### 3.2. Evaluation of SMN Protein Production

SMN protein is considered to be unstable to repeated freeze–thaw cycles [20]. To avoid the influence of freeze–thawing, cell lysates were analyzed after a single cycle.

**Figure 3:**
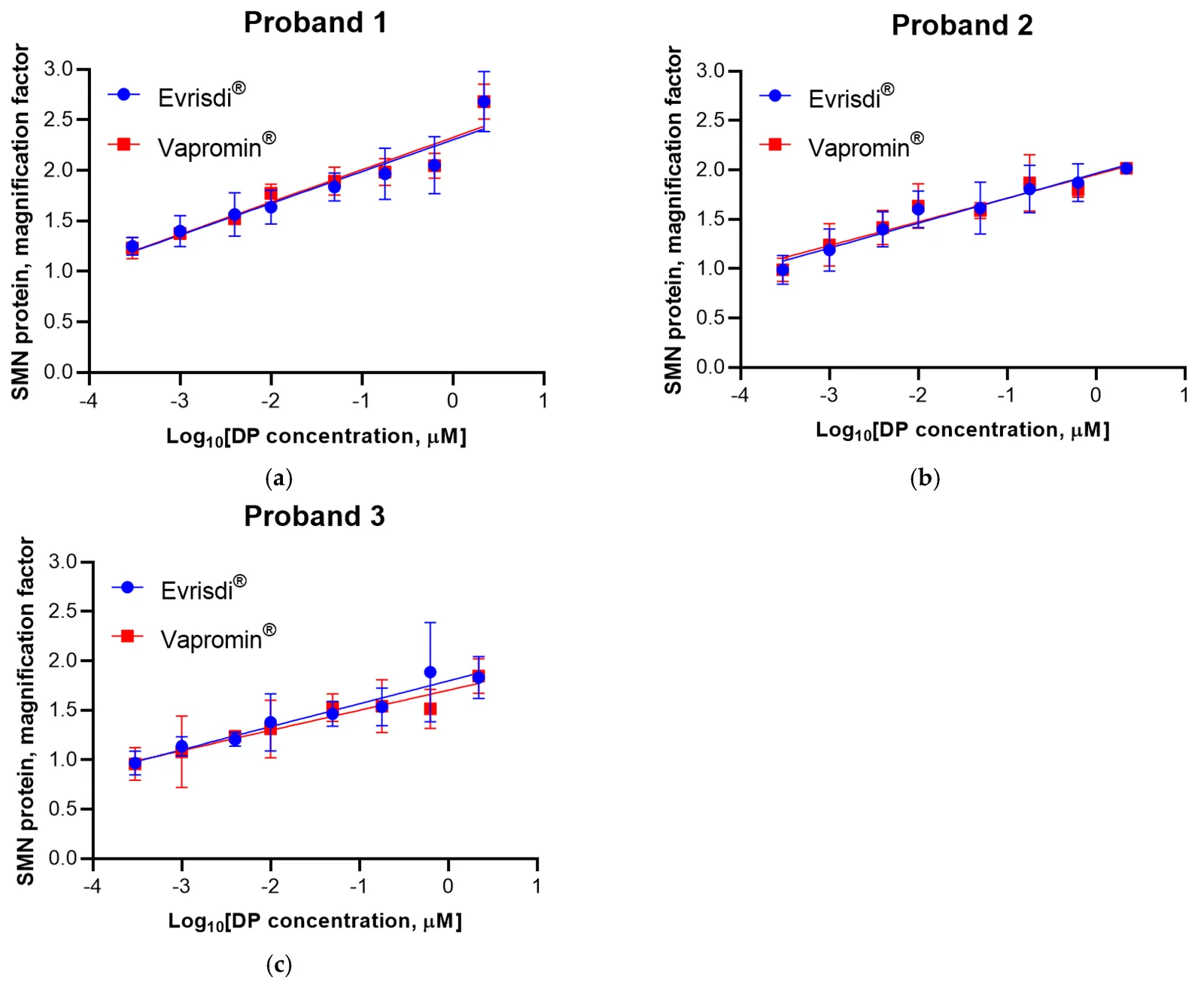
Plots of SMN protein levels versus each drug log-concentration in fibroblasts from three SMA probands (**a**–**c**). SMN protein levels are expressed as fold changes relative to the baseline level in fibroblasts from SMA probands without drug. Mean levels and standard deviations (based on triplicates) are shown for each drug concentration.

Fibroblasts from three SMA probands were exposed to increasing concentrations (0.0003 to 2.2 µM) of risdiplam preparations (either Evrysdi^®^ (Roche, Basel, Switzerland) or Vapromin^®^), followed by relative SMA protein level measurement, expressed as fold changes relative to the baseline level in fibroblasts from SMA probands without drugs (Figure 3). Linear positive trends were established versus each drug log-concentration.

Using the F-test for extra sum-of-squares, a significant difference of slopes from zero and the absence of significant differences in parameters between drugs for each proband were shown (Table 3).

The maximum increase in SMN protein levels was observed after treatment with 2.2 µM of the drug. The SMN protein level increased from 2 to 2.6 times (average value for three batches) compared to untreated fibroblasts.

These findings support the conclusion that Evrysdi^®^ (Roche, Basel, Switzerland) and Vapromin^®^ have similar effects on SMN protein production in fibroblasts from SMA donors.

### 3.3. Immunoblotting

**Figure 4:**
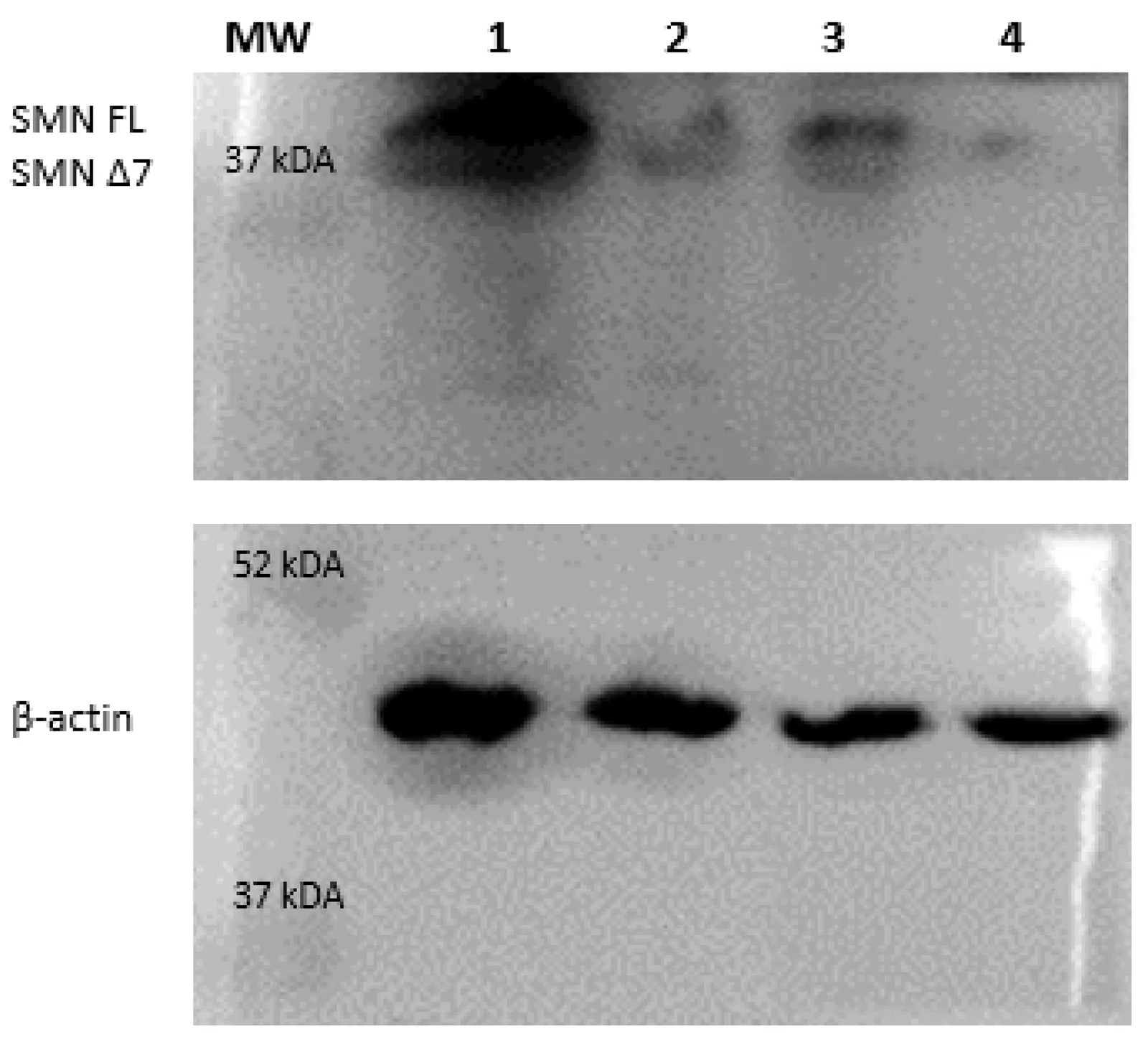
Western blot analysis of SMN protein expression in fibroblasts. The membrane was cut into two sections and probed with a monoclonal anti-SMN antibody and a monoclonal anti-β-actin antibody as a loading control. Lanes: 1, lysate from healthy donor fibroblasts; 2, lysate from SMA patient fibroblasts; 3, SMA patient fibroblasts treated with 2.2 µM Vapromin^®^; 4, SMA patient fibroblasts treated with 0.02 µM Vapromin^®^.

Western blot analysis confirmed the presence of full-length SMN2 protein in fibroblasts derived from patients with SMA following treatment with 2.2 µM risdiplam. Immunoblotting with anti-SMN monoclonal antibodies revealed two distinct bands, likely corresponding to the two major isoforms of SMN protein. These findings are consistent with previously published reports [21,22] (Figure 4).

### 3.4. Cell Viability

**Figure 5:**
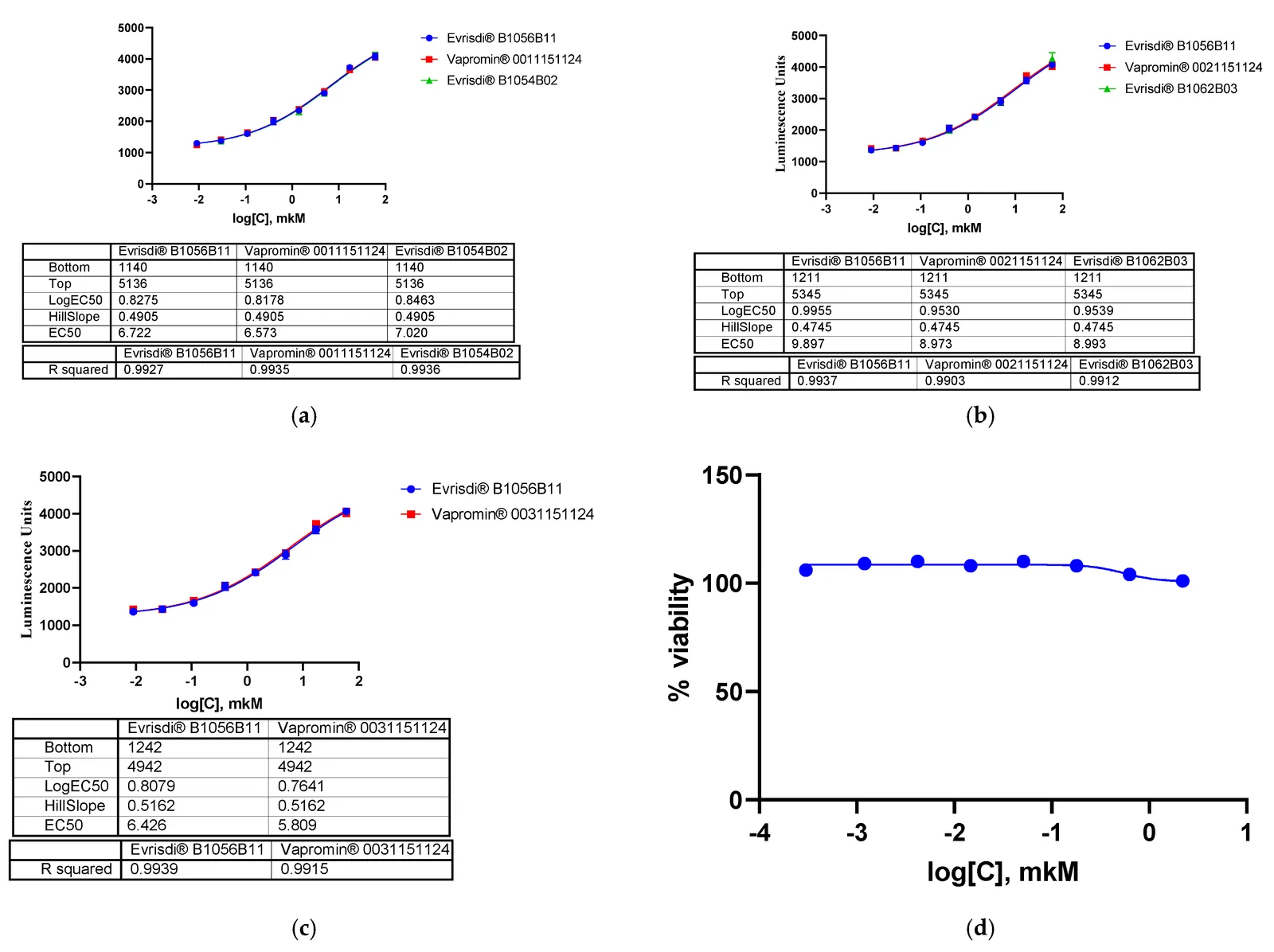
Plots showing the activity of different batches of risdiplam on the reporter cell line and cytotoxicity. (**a**–**c**): Each dot represents different drug log-concentrations versus responses of the reporter cell line in luminescence units. (**d**): Results of the XTT cytotoxicity assay following the treatment of fibroblasts with Vapromin^®^ at concentrations ranging from 0.3 nM to 2.2 μM.

Cell viability was routinely assessed via visual inspection using light microscopy. Cell metabolic activity was assessed to determine whether the tested concentrations of risdiplam affected cell viability. The XTT assay demonstrated that treatment with risdiplam at concentrations ranging from 0.3 nM to 2.2 μM had no significant effect on the viability of fibroblast cells, indicating the absence of cytotoxicity within the tested concentration range (Figure 5d).

### 3.5. Results of Determination of Exon 7 Inclusion Efficiency Using the CHO SMN2-LUC MP 4G6 Reporter Cell Line

Addition of risdiplam to the reporter cell line CHO SMN2-LUC MP 4G6 led to a dose-dependent response (Figure 5).

The activity of each sample was calculated compared to the RS. The results are presented in Table 4.

**Figure 6:**
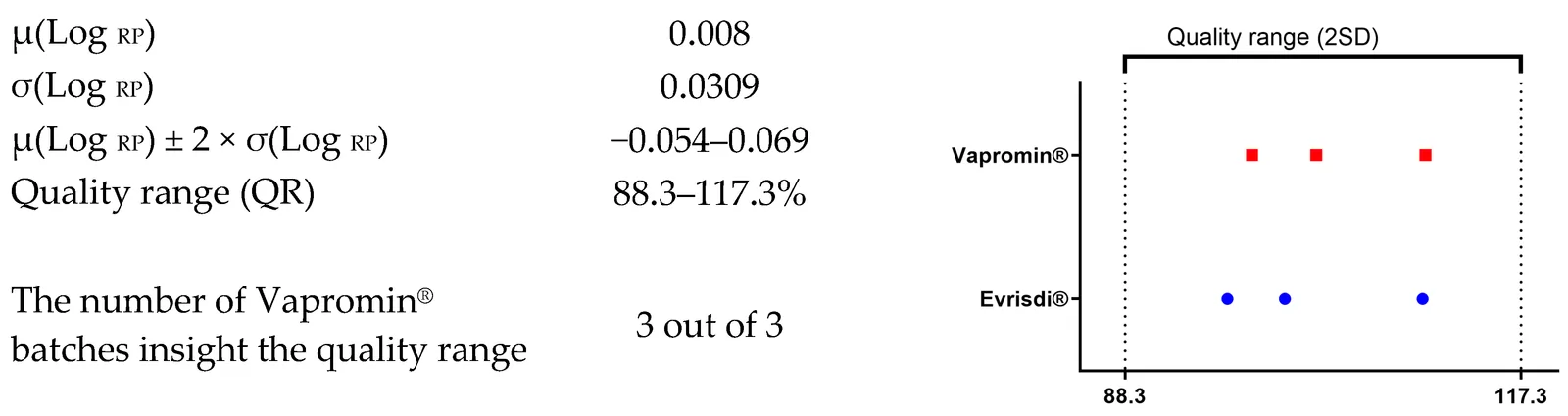
Comparison of individual values obtained for Vapromin^®^ with the quality range (2SD) of Evrysdi^®^ (Roche, Basel, Switzerland).

The individual values of relative specific activity for Vapromin^®^ were compared with the QR (2SD) of Evrysdi^®^ (Roche, Basel, Switzerland, Figure 6).

The statistical analysis of exon 7 inclusion during mRNA splicing of the *SMN2* gene on a reporter culture showed that the values of specific activity obtained for all batches of Vapromin^®^ were within the QR of Evrysdi^®^ (Roche, Basel, Switzerland).

## 4. Discussion

Despite the extensive clinical and preclinical use of DMTs, there is currently no standardized or broadly accepted in vitro framework for evaluating the efficacy of low-molecular-weight or antisense oligonucleotides targeting *SMN2* splicing. Published studies frequently employ different cellular models, cell-seeding densities, and transfection strategies, often selected empirically and without systematic comparison [23].

In the present study, we compared the biological activity of the generic drug Vapromin^®^ and its reference drug Evrysdi^®^ (Roche, Basel, Switzerland)—the first approved orally administered low-molecular-weight drug for the therapy of SMA. Risdiplam distributes throughout both the central and peripheral nervous system and modulates splicing of *SMN2* pre-mRNA toward the production of full-length mRNA to increase the levels of functional SMN protein [24]. Although it is generally not necessary to compare the in vitro biological activity of generic drugs, the clinical significance of risdiplam makes such comparison favorable.

Several assays are available to measure the biological function of SMN protein [25,26,27]. Primary fibroblasts are particularly suitable for this purpose, since they retain the genetic and epigenetic characteristics of the donor while being homogeneous and reproducible [28,29].

To assess the effectiveness of *SMN2* pre-mRNA splicing and the amount of SMN protein produced, we used fibroblasts isolated from SMA probands with a homozygous deletion of exons 7–8 of the *SMN1* gene and different numbers of the *SMN2* gene copies.

Analyzing the obtained results for the relative transcription levels of *SMN2* FL and *SMN2* Δ7, we noted that, regardless of the drug (Evrysdi^®^ (Roche, Basel, Switzerland) or Vapromin^®^), the level of *SMN2* FL transcripts in fibroblasts of each proband changed in a concentration-dependent manner.

Signoria et al. studied the influence of the *SMN2* copy number, age, and gender of the donor on the relative level of *SMN2* FL transcription when treating patient fibroblasts with risdiplam and the antisense oligonucleotide nusinersen. The most considerable, however, the most variable change in the relative level of transcription was observed in patients with three copies of the *SMN2* gene [29]. This may explain the results obtained in our study, since we used fibroblasts from patients with both three (probands 1 and 2) and four copies of the SMN gene (proband 3).

We also found that, at the maximum risdiplam concentration of 2.2 μM, the level of *SMN2* FL transcripts was the same as the level of SMN FL in fibroblasts treated with 0.18 μM risdiplam. A similar effect was observed by other researchers when studying a saturating dose of antisense oligonucleotides [30]. The authors suggest that high concentrations of ASO targeting Intronic Splicing Silencer N1 promote the activation of the cryptic splicing site in intron 6 of the *SMN2* gene.

Risdiplam enters cells primarily by passive diffusion due to its high lipophilicity, which allows it to cross cell membranes throughout the body, including the blood–brain barrier. Transcriptional changes should be confirmed by protein-level investigations, since changes in SMN expression levels may not be accompanied by effective changes in stable protein production, as the dynamics and half-lives of mRNA and proteins may not be comparable [3]. Using primary SMN donors’ fibroblasts, we characterized the *in vitro* response to *SMN2* splicing modification risdiplam and demonstrated that Vapromin^®^ caused a dose-dependent increase in SMN protein levels similar to Evrysdi (Roche, Basel, Switzerland).

Changes in SMN protein production in fibroblasts were also established, which is consistent with literature data. SMN protein levels have been studied in many cell types [27]. Notably, significant differences in SMN protein levels were found in platelets, erythrocytes, and PBMCs, highlighting the importance of investigating tissue-specific SMN expression [20,31]. It is considered that SMN protein levels in fibroblasts from healthy donors are several times higher than in SMA probands. Moreover, SMN protein production levels increase by 50% or more upon treatment of fibroblasts with mRNA splicing-correcting drugs [25,31]. Using imaging flow cytometry, it was shown that fibroblasts from SMA patients expressed SMN protein at 20–30% of normal levels [32]. A correlation was also established between SMN protein levels and the *SMN2* copy number in fibroblasts, as well as with clinical characteristics, indicating that fibroblasts isolated from the skin of SMA carriers may be a reliable cell type for SMN biomarker studies. The lack of correlation between SMN protein levels in PBMCs and fibroblasts likely indicates differences between these tissues [27].

The CHO SMN2-LUC MP 4G6 reporter cell line was constructed to assess exon 7 inclusion during *SMN2* mRNA splicing. It triggers the relevant signaling pathway in response to specific activation and allows visualization of the drug’s mechanism of action *in vitro*. This cell line could be used in a robust, sensitive, and reproducible *in vitro* assay for evaluating the efficacy of splicing modifiers targeting *SMN2* splicing.

The cell line was used to determine the specific activity of each batch. The “quality range” method (formulas are given above) was also used for statistical analysis. The individual values obtained for the Vapromin^®^ batches fell within the QR established for Evrysdi^®^ (Roche, Basel, Switzerland).

## 5. Conclusions

Therefore, these data experimentally confirm that the *in vitro* biological activity of the generic drug Vapromin^®^ is comparable to that of Evrysdi^®^. However, further investigation should be performed on a large number of batches to show equivalence testing (TOST).

## Figures and Tables

**Table 1:** Comparison of *SMN2* FL mRNA transcription level curves in fibroblasts from three SMA probands.

Proband	DP	Spearmen’s r	*p*-Value for Correlation	AUC	SE of AUC	AUC Diff.	*p*-Value for AUC Diff.
1	Evrysdi^®^	0.8571	0.0107	4.688	0.3404	0.137	0.7438
	Vapromin^®^	0.8333	0.0154	4.551	0.2446		
2	Evrysdi^®^	0.8333	0.0154	5.254	0.4005	0.581	0.3345
	Vapromin^®^	0.8333	0.0154	4.673	0.4494		
3	Evrysdi^®^	0.9048	0.0046	5.199	0.3888	0.378	0.4371
	Vapromin^®^	0.8333	0.0154	4.821	0.2923		

**Table 2:** Comparison of *SMN2* Δ7 mRNA transcription level curves in fibroblasts from three SMA probands.

Proband	DP	Spearmen’s r	*p*-Value for Correlation	AUC	SE of AUC	AUC Diff.	*p*-Value for AUC Diff.
1	Evrysdi^®^	−0.9940	0.0001	1.514	0.131	0.011	0.9506
	Vapromin^®^	−0.9701	0.0006	1.503	0.119		
2	Evrysdi^®^	−0.9762	0.0004	2.000	0.160	0.227	0.3227
	Vapromin^®^	−0.9524	0.0011	1.773	0.164		
3	Evrysdi^®^	−0.9999	0.0001	2.221	0.171	0.067	0.7117
	Vapromin^®^	−0.9999	0.0001	2.154	0.059		

**Table 3:** Comparison of linear trends for SMN protein level in fibroblasts from three SMA probands.

Proband	DP	Slope	Intercept	R Square	*p*-Value for Slopes Non-Zero	*p*-Value for Slopes Diff.	*p*-Value for Intercepts Diff.
1	Evrysdi^®^	0.3145	2.302	0.7638	0.0001	0.9035	0.7857
	Vapromin^®^	0.3198	2.326	0.8702	0.0001		
2	Evrysdi^®^	0.2518	1.964	0.7613	0.0001	0.7564	0.8631
	Vapromin^®^	0.2399	1.954	0.7717	0.0001		
3	Evrysdi^®^	0.2315	1.797	0.6469	0.0001	0.5388	0.4078
	Vapromin^®^	0.2031	1.703	0.6354	0.0001		

**Table 4:** Relative specific activity on the reporter cell culture.

Sample	Batch	%-Activity	Geometric Mean	% GSD
Evrysdi^®^	B1056B11	100.0	101.8%	7.4%
	B1054B02	95.8		
	B1062B03	110.1		
Vapromin^®^	0011151124	102.3	103.3%	6.3%
	0021151124	110.3		
	0031151124	97.6		

## Data Availability

The original contributions presented in this study are included in the article. Further inquiries can be directed to the corresponding author.

## Abbreviations

The following abbreviations are used in this manuscript:

AUC	Area under curve
CHO	Chinese Hamster Ovary
DP	Drug product
DMTs	Disease-modifying therapies
EMA	European Medicines Agency
ECL	Enhanced chemiluminescence
FL	Full length
FDA	Food and Drug Administration
HRP	Horseradish peroxidase
JSC	Joint-Stock Company
mRNA	Messenger ribonucleic acid
MLPA	Multiplex ligation-dependent probe amplification
RS	Reference standard
PBS	Phosphate-buffered saline
PBST	Phosphate-buffered saline containing tween 20
PD	Pharmacodynamics
PK	Pharmacokinetics
PVDF	Polyvinylidene fluoride
QR	Quality range
RSA	Relative specific activity
SD	Standard Deviation
SMA	Spinal muscular atrophy
SMN	Survival motor neuron
TBST	Tris-buffered saline containing Tween 20
TOST	Two One-Sided Tests
